# Spatial Analysis of Infections by *Toxoplasma gondii* and *Neospora caninum* (Protozoa: Apicomplexa) in Small Ruminants in Northern Italy

**DOI:** 10.3390/ani9110916

**Published:** 2019-11-04

**Authors:** Alessia Gazzonis, Luca Villa, MariaTeresa Manfredi, Sergio Zanzani

**Affiliations:** Department of Veterinary Medicine, Università degli Studi di Milano, 20133 Milan, Italy; alessia.gazzonis@unimi.it (A.G.); luca.villa@unimi.it (L.V.); mariateresa.manfredi@unimi.it (M.M.)

**Keywords:** parasites, protozoa, Apicomplexa, *Toxoplasma gondii*, *Neospora caninum*, goat, sheep, spatial analysis, Italy

## Abstract

**Simple Summary:**

*Toxoplasma gondii* and *Neospora caninum* are among the major abortifacient pathogens in sheep and goats. Environmental risk factors may contribute to the spread of both protozoans among sheep and goats. In this study, the spatial analysis provided additional information useful to study the patterns of distribution and spread of diseases, increasing the comprehension of the association between disease processes and explanatory environmental variables. The research aimed to explore whether geographical or environmental factors could influence the infections by *T. gondii* and *N. caninum* in sheep and goats in northern Italy and identify areas at risk of infection. A heterogenic distribution of seroprevalence both pathogens was highlighted in the study area with areas at high or low risk according to the protozoan. Particularly, annual temperature, rainfall, and their association enhanced the risk of *T. gondii* and *N. caninum* infection among sheep. Otherwise, the risk for goats to acquire those infections did not depend on environmental or geographical features, but instead on factors associated with individual characteristics or farm management. The results observed in this study suggest spatial analysis is a useful tool to implement control measures to prevent these important protozoan diseases for small ruminant breeding.

**Abstract:**

The objectives of this study were: (i) To investigate possible geographical or environmental factors influencing the infections by *Toxoplasma gondii* and *Neospora caninum* in sheep and goats in northern Italy; (ii) to identify areas at risk of infection to set up preventive measures. Forty-three sheep and goat farms were included. Their locations were plotted and associated with *T. gondii* and *N. caninum* seroprevalence, then the distribution of farms’ prevalence was evaluated by spatial analysis. Significant clusters for both low and high prevalence were obtained, and a generalized linear model with ordinal logistic regression was implemented to verify if spatial clustering could be due to climate factors (temperature, rainfall, and their interaction). Clusters of high (80.0%) and low prevalence (28.12%) resulted for *T. gondii* seroprevalence in sheep farms. No significant clusters resulted for goat farms. Clusters of high (38.68%) and low prevalence (21.23%) resulted for *N. caninum* seroprevalence in sheep farms. One high-prevalence cluster (15.62%) resulted for goat farms. For goats, spatial analysis and analysis on climatic data showed the absence of environmental significant risk factors associated with *T. gondii* or *N. caninum* infection. On the contrary, for sheep, annual temperature, rainfall, and their association affected the risk of *T. gondii* and *N. caninum* infection. Particularly, high temperatures and abundant rainfalls were related to *T. gondii* seroprevalence, while low temperatures and scarce rainfalls were related to *N. caninum* seroprevalence.

## 1. Introduction

*Toxoplasma gondii* is considered a major foodborne parasite and poses significant risks to public health. In Europe, 1.48 cases of congenital infection per 100,000 live births were registered in 2013–2016 [1], with seroprevalence values in pregnant women up to 60% [2]. If acquired during pregnancy, *T. gondii* infection, which is usually asymptomatic in immunocompetent patients, may have severe repercussions on the fetus (i.e., chorioretinitis, hydrocephalus, abortion) [3]. Also, toxoplasmosis may cause encephalitis, pneumonitis, and myocarditis in patients with a compromised immune system [4].

Several routes of infection exist in humans, with the most common route of infection being through the consumption of food products of animal origin [5]. Particularly, small ruminants’ meat is considered one of those containing the highest number of tissue cysts, and therefore a major source of infection as a consequence of poor food hygiene practices and/or undercooking of meat, especially in those countries where the consumption of sheep and goat meat is part of the culinary tradition [6].

Furthermore, *T. gondii*, together with the closely related *Neospora caninum*, is also a relevant issue for small ruminants’ breeding, as they are among the major abortifacient pathogens in sheep and goats [7]. Both pathogens are widespread in these species, showing a wide range of seroprevalence values varying among different countries and depending on the breeding conditions [8,9,10,11,12,13,14,15]. Various risk factors linked to the farm management have been highlighted, such as the extensive rearing system with access to pasture and the size of the farm [10,13,14,15].

However, at the same or similar breeding conditions, there may be differences among countries or regions in the spread of T. *gondii* and *N. caninum* infections within small ruminant populations [16]. In fact, environmental risk factors, modulating the spread of pathogens and influencing the physiology and behavior of hosts [17], may contribute to the spread of both protozoans among sheep and goats, given the predominance in these species of horizontal transmission through the ingestion of environmental oocysts. Vice-versa, small ruminants may serve as indicators of the spread of these pathogens in the environment. For this purpose, spatial analysis provides additional information useful to study and interpret the patterns of distribution and spread of diseases, increasing the comprehension of the association between disease processes and explanatory environmental variables.

Particularly in parasitology, spatial techniques and the analysis of climatic data found applications in the study of zoonotic parasitic diseases (i.e., cystic echinococcosis, onchocerciasis, fasciolosis, schistosomiasis) or in the studies of vector-borne diseases [18,19,20,21,22,23].

Considering *T. gondii* and *N. caninum* infection in animals, few epidemiological studies have been carried out utilizing geospatial tools [16,24,25,26,27]. Therefore, epidemiological data on *T. gondii* and *N. caninum* infections in sheep and goats in northern Italy were submitted to geospatial analysis, and climatic data were considered and analyzed. The study area (Lombardy region, northern Italy) was selected, since several management systems in sheep and goat breeding are represented in territories varying from the plains to the mountains. As several epidemiological studies demonstrated the presence of *T. gondii* and *N. caninum* in domestic and wild animals including small ruminants from this territory [14,15,28,29,30,31,32], the present study aimed to investigate possible geographical or environmental factors influencing the distribution of these protozoal infections and to identify areas at high risk of infection in order to establish preventive measure to control their spread.

## 2. Materials and Methods

*T. gondii* and *N. caninum* farms seroprevalence were determined in previous epidemiological studies [14,15]. In the present study, 414 goats and 428 sheep from 43 farms from Varese, Milano, and Bergamo provinces (Lombardy, northern Italy) were included. Of the 43 farms, 14 farms reared both sheep and goats. The minimum sample size was calculated based on data on small ruminants’ population in Lombardy, provided by the National Zootechnical Database (data available at https://www.vetinfo.sanita.it/). In the province of Milan (southern area), there were 7153 small ruminants (27 sheep and 47 goat farms), 49,218 (89 sheep and 365 goat farms) in Bergamo (eastern area), and 9238 (109 sheep and 186 goat farms) in Varese (western area). The minimum sample size (385 goats and 385 sheep) was determined using the program Winepiscope 2.0 (http://www.clive.ed.ac.uk/winepiscope/0) to exclude (in case of all negative samples) a *T. gondii* and *N. caninum* seroprevalence of ≤ 50% within the animals at a confidence level of 95%and with a statistical error of 5%. The individual presence of antibodies anti-*T. gondii* and anti-*N. caninum* was used to determine the *T. gondii* and *N. caninum* farms seroprevalence.

An individual *T. gondii* seroprevalence of 59.3% and 41.7% was recorded in sheep and goats, respectively, while 19.3% of sheep and 5.7% of goats showed antibody anti-*N. caninum*. Concerning *T. gondii*, 87.5% and 96.6% of ovine and caprine farms, respectively, included in the studies showed at least one positive animal, while 89.4% and 32.1% of sheep and goat farms, respectively, scored positive for *N. caninum* infection. Risk factor analysis was performed in both studies. Rearing system and farm size were shown to enhance the risk of infection by both pathogens and in both host species.

Farms’ locations were plotted using Google Earth Pro ver.7.3 (Google LLC, Mountain View, California) to create a KML file that was then uploaded in the free online tool software Kml2x (http://www.zonums.com/online/kml2x/, Zonum Solutions, Tucson, Arizona) to obtain the farms’ UTM zone 32N coordinates on the WGS84 Datum. Farms’ locations were then associated with *T. gondii* and *N. caninum* prevalence of infection (seropositivity).

**Figure 1:**
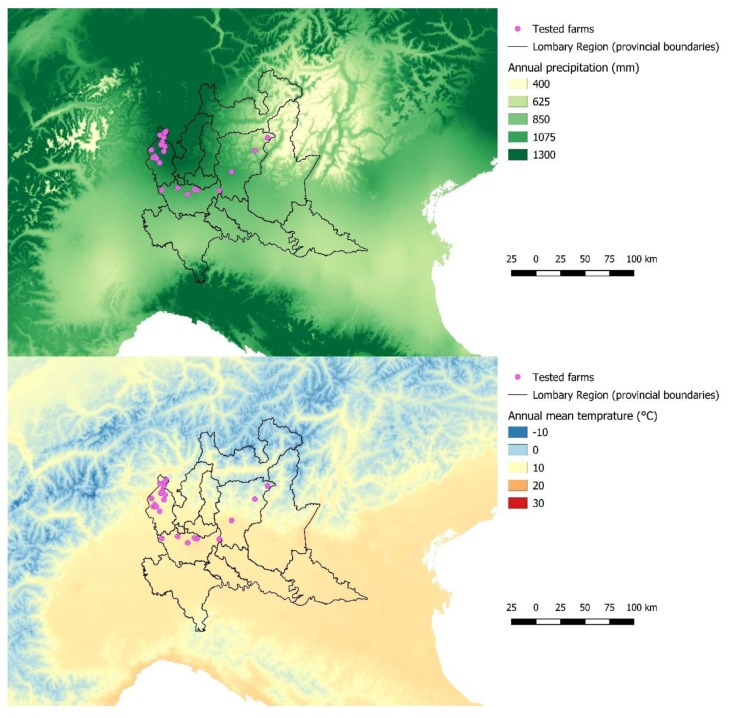
Spatial distribution of surveyed small ruminant farms in the study area according to temperature (upper) and rainfall maps (lower).

To evaluate whether the farms’ prevalence was unevenly distributed in the study area, spatial analysis with a weighted normal model was performed using SaTScan ver. 9.3.1 [33,34]. The purely spatial scan statistic was defined by a circular or elliptic window. The window imposed on the map was, in turn, centered on each of several possible grid points positioned throughout the study region. For each grid point, the radius of the window varied continuously in size from zero. An upper limit of 50% of the included farms was specified. In this way, the circular or elliptic window was flexible in both location and size. In total, the method created an infinite number of distinct geographical windows with different sets of neighboring data locations within them. Each circle or ellipse was a possible candidate cluster. To test whether the prevalence of infection was randomly distributed or there was a major or minor prevalence within the window, a likelihood ratio test was performed for each window basing on observed and expected prevalence inside and outside the window. The *p*-value of the maximum likelihood ratio test statistic was obtained after 999 Monte Carlo replications, and only statistically significant clusters (*p* < 0.05) were considered [33]. Farm size and management can affect *T. gondii* and *N. caninum* prevalence in small ruminants [14,15]. So, to include an adjustment for size and management, they were introduced as covariates in the case file. Farms’ locations were also introduced in a Geographical Information System using QGIS ver. 3.6 (Quantum GIS Development Team, www.qgis.org) to couple each farm with meteorological data. Raster layers with annual mean temperatures (BIO1, expressed in tenths of °C) and annual rainfalls (BIO12, expressed in mm) were obtained from WorldClim ver. 2.0 (http://www.worldclim.org, Feed the Future Innovation Lab for Collaborative Research on Sustainable Intensification, Kansas State University, Manhattan, Kansans) with a resolution of 30 arc-second (~1 km). Climate data were expressed as mean monthly values averaged over 30 years (1970–2000). The spatial distribution of tested farms in temperature and rainfall maps is shown in Figure 1.

When spatial analysis by SaTScan produced significant clusters for both low and high prevalence, farms sited inside low-prevalence clusters were scored 0, farms outside clusters were scored 1, and farms sited inside high-prevalence clusters were scored 2. The scores were introduced as dependent variables in generalized linear models with ordinal logistic regression. Temperature, rainfall, and their interaction were introduced as independent variables to verify if spatial clustering could be due to climate factors. If only low- or high-prevalence clusters were produced, farms were scored 0 (outside the clusters) and 1 (inside), and a generalized linear model with binary logistic regression was implemented. Independent variables were tested for multicollinearity by tolerance and variance inflation factor; final models were obtained by backward elimination of not significant independent variables. Statistical analyses were performed using SPSS ver. 20 (IBM, Chicago, IL, USA).

## 3. Results

### 3.1. Spatial Analysis

**Figure 2:**
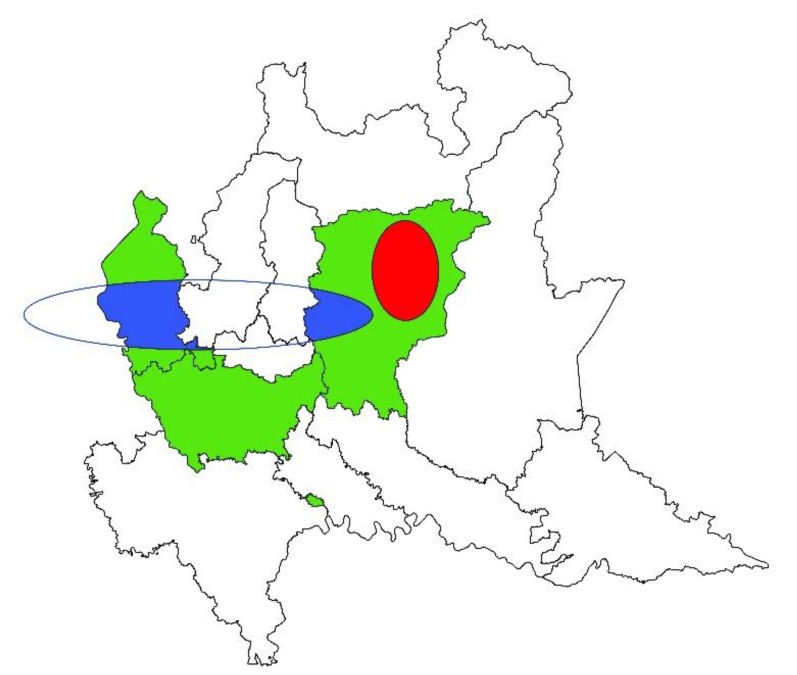
Geographical allocation of significant clusters of *Toxoplasma gondii* infections in sheep farms in northern Italy. Red represents the cluster of high prevalence of infection, blue represents the cluster of low prevalence of infection, and green represents the studied area (Varese, Milano, and Bergamo provinces).

The spatial analysis highlighted a heterogenic distribution of *T. gondii* prevalence of infection among sheep farms, creating one significant cluster of high prevalence and one of low prevalence (Figure 2). Inside the high prevalence elliptic cluster (*p*-value = 0.001; center: 45.87558976 N, 9.89011637 E; semi-minor axis = 11.23 km; semi-major axis = 16.85 km) the weighted farm prevalence of infection was 80.0%, while outside it was 46.12%. Inside the low-prevalence cluster (*p*-value = 0.001; center: 45.74440663 N, 8.99019130 E; semi-minor axis = 11.77 km; semi-major axis = 58.85 km) the weighted farm prevalence of infection was 28.12%, while outside it was 61.90%. No significant clusters of *T. gondii* prevalence of infection resulted for goat farms.

**Figure 3:**
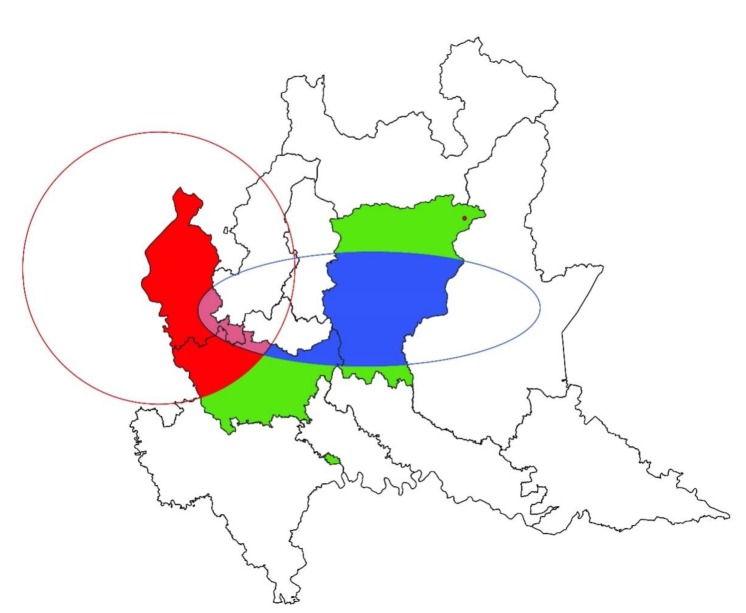
Geographical allocation of significant clusters of *Neospora caninum* infections in sheep farms in northern Italy. Red represents the cluster of high prevalence of infection, blue represents the cluster of low prevalence of infection, and green represents the studied area (Varese, Milano, and Bergamo provinces).

**Figure 4:**
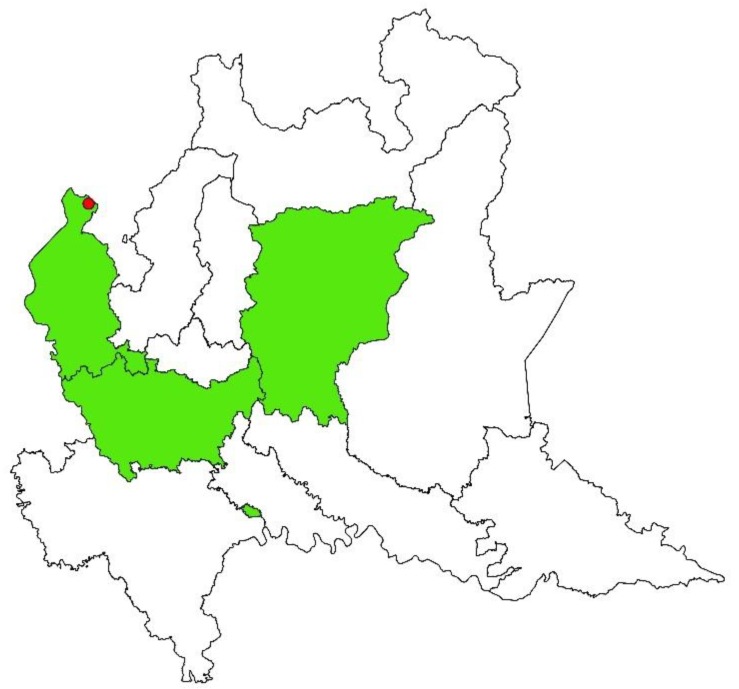
Geographical allocation of the significant cluster (in red) of *Neospora caninum* high prevalence in goat farms in northern Italy. Green represents the studied area (Varese, Milano, and Bergamo provinces).

The spatial distribution of *N. caninum* prevalence of infection was heterogeneous both in sheep and goat farms (Figure 3 and Figure 4, respectively). Concerning sheep farms, SatSscan statistic showed two high-prevalence and one low-prevalence clusters. The two high-prevalence clusters were both circular. The first (*p*-value = 0.003; center: 45.84157841 N, 8.63720649 E) was larger and presented a radius of 52.37 km, while the second (*p*-value = 0.009; center: 46.00911941 N, 10.15571785 E) presented a radius of 1.10 km. Inside the first cluster, the *N. caninum* weighted prevalence was 38.68%. Outside the first cluster, the prevalence was 13.04%. Weighted farm prevalence inside the second cluster was 21.23%, which was significantly higher than outside (18.98%). The low-prevalence cluster of sheep farms was elliptic (*p*-value = 0.009; center: 45.69829573 N, 9.67727186 E; semi-minor axis = 21.95 km; semi-major axis = 65.87 km). Inside it, the weighted prevalence of infection was 8.82%, while outside it was 26.36%. There were no farms in the overlapping area. Spatial analysis of *N. caninum* prevalence of goat farms showed only one high-prevalence cluster (*p*-value = 0.002). It was circular (center: 46.07538855 N, 8.81704986 E; radius = 1.81 km) and comprised three farms within it. The weighted farm prevalence was 15.62% and 2.55% inside and outside it, respectively.

### 3.2. Risk Factors Analysis

**Figure 5:**
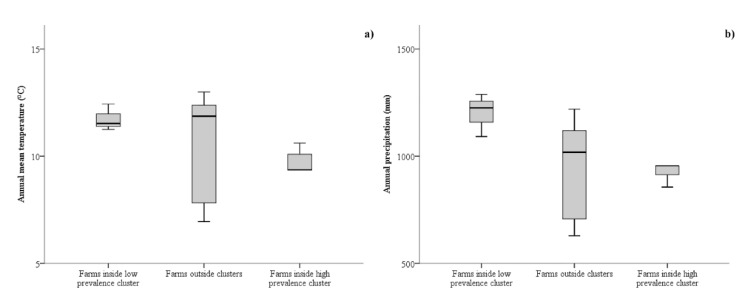
Annual mean temperature (**a**) and annual rainfall (**b**) in sheep farms sited inside the low *T. gondii* prevalence cluster, outside clusters, and inside the high *T. gondii* prevalence cluster.

The spatial analysis by SaTScan produced significant clusters for both low and high *T. gondii* prevalence in sheep farms, so a generalized linear model with ordinal logistic regression was implemented. Both univariate and multivariate multilevel analyses were implemented (Table 1 and Table 2, respectively). In the multivariate analysis, the interaction of the two climatic variables was introduced. The annual mean temperature and annual rainfall of farm observed in the low-prevalence cluster, outside clusters, and inside the high-prevalence cluster are shown in Figure 5. In the final multivariate multilevel analysis, all the introduced independent variables were significant risk factors (Table 2). Higher *T. gondii* prevalence in sheep farms was positively related to both annual mean temperature (*p*-value = 0.010; OR: 2.271; 95% CI: 1.219–4.232) and annual rainfall (*p*-value = 0.009; OR: 1.093; 95% CI: 1.022–1.169). Interaction between the two climatic variables was negatively related to *T. gondii* prevalence (*p*-value = 0.007; OR: 0.9991; 95% CI: 0.9984–0.9997). No significant clusters were obtained from the spatial analysis of *T. gondii* prevalence in goat farms, so risk factor analysis was not implemented.

**Figure 6:**
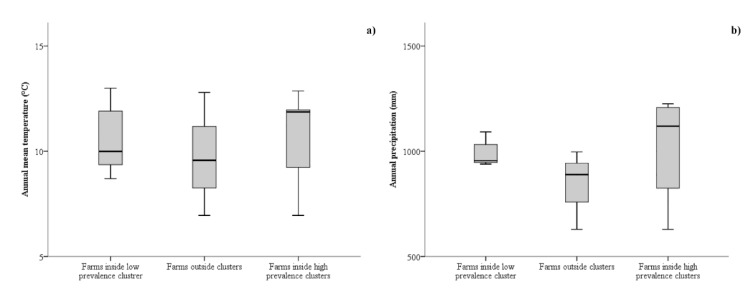
Annual mean temperature (**a**) and annual rainfall (**b**) in sheep farms sited inside the low *N. caninum* prevalence cluster, outside clusters, and inside the high *N. caninum* prevalence clusters.

The detection of both low and high *N. caninum* prevalence clusters in sheep farms allowed the risk factor analysis by a generalized linear model with ordinal logistic regression. Both univariate and multivariate multilevel analyses were implemented (Table 3 and Table 4, respectively). In the multivariate analysis, the interaction of the two climatic variables was introduced. The annual mean temperature and annual rainfall of farm observed inside the low-prevalence cluster, outside clusters, and inside the high-prevalence cluster are shown in Figure 6. The final model of the multivariate multilevel analysis included all the considered independent variables (Table 4). Higher *N. caninum* prevalence in sheep farms was negatively related to both annual mean temperature (*p*-value = 0.005; OR: 0.567; 95% CI: 0.381–0.844) and annual rainfall (*p*-value = 0.010; OR: 0.947; 95% CI: 0.908–0.987). Interaction between the two climatic variables was positively related to *N. caninum* prevalence (*p*-value = 0.005; OR: 1.0006; 95% CI: 1.0002–1.001).

The spatial analysis by SaTScan produced only a significant cluster for high *N. caninum* prevalence in goat farms, so a generalized linear model with binary logistic regression was implemented. None of the possible meteorological risk factors introduced in the model was significant in univariate or multivariate analysis (Table 5).

## 4. Discussion

The current study provided the spatial and climatic analyses on serological data on *T. gondii* and *N. caninum* infection in small ruminants bred in northern Italy, aimed at highlighting environmental features enhancing the risk for animals to become infected. The identification of areas at high risk of infection allowed us to map the environmental spread of the considered pathogen and to eventually set up control measures.

For goats, spatial analysis and analysis on climatic data showed the absence of environmental significant risk factors associated with *T. gondii* or *N. caninum* infection, emphasizing that those infections did not depend on environmental or geographical features but more on factors related to individual characteristics or farm management. On the contrary, the binary logistic regression analysis revealed that annual temperature, rainfall, and their association enhanced the risk of *T. gondii* infection in sheep. Concerning *N. caninum* in sheep, temperature and rainfall showed influence on the spread of *N. caninum*, being it higher when the climatic factors analysed have lower values. These results indicated that there were differences between *T. gondii* and *N. caninum* diffusion in the ovine species, and differences in the environmental survival of the oocysts of the two pathogens could exist. Indeed, mild temperatures have been suggested to enhance *N. caninum* seroprevalence values [25,35], as well as the risk of abortions in cattle [36], since they are supposed to be optimal for *N. caninum* oocysts sporulation [37]. Concerning *T. gondii*, their oocysts seems to be more resistant than those of *N. caninum*, since environmental unsporulated oocysts are proven to lose their capacity to sporulate and thus to be infective after freezing (24 h at −21 °C or 7 days at −6 °C) or heating (50 °C for 10 min) [38].

Further, according to spatial analysis, clusters at high and low risk of infection that were different and opposed for *T. gondii* and *N. caninum* were depicted for the ovine species. Particularly, as demonstrated by Figure 1, the eastern part of the study area, where a cluster of high risk of *T. gondii* infection and one at low risk of *N. caninum* infection for sheep resulted, could be more favorable to the survival of *T. gondii* oocysts, characterized to lower temperatures than the western part. In both sheep and goats, a cluster of high prevalence of *N. caninum* infection was detected in the western part of the study area, showing that this area could be more suitable for this protozoan than the eastern one. Other environmental factors—not strictly linked to geographic or climatic characteristics—can play a role in the spread of infections, such as anthropization. Indeed, in a certain area, a high human density corresponds to a high density of cat and dog populations. Cat and dog populations are definitive hosts of *T. gondii* and *N. caninum*, respectively, which may influence and modulate the spread of pathogens in the environment and therefore in intermediate hosts [26,39].

Furthermore, additional risk factors that were not considered in the present study may help explain the differences in the environmental distribution of *T. gondii* and *N. caninum*. Additional risk factors include the use of natural water sources for watering animals or grazing enhancing the risk of *T. gondii* infection and the presence in the vicinity of other farms or herds, or the presence of wildlife with which sheep and goats can come into contact are among the variables capable of influencing the risk of *T. gondii* and *N. caninum* infections [13,14,15]. Particularly, wildlife could play a role in the horizontal transmission of *N. caninum* in the study area. Despite the fact that the role of red foxes as possible definitive hosts of *N. caninum* is still a hypothesis [40], previous authors showed that seroprevalence in beef calves was spatially associated to abundance of wild canids [41], and fox control measures were found to be protective against infection with *N. caninum* in beef herds [42]. The possibility that the clusters of *N. caninum* obtained in the present study may also be influenced by the spatial distribution of wild fauna should be considered, thus reducing the real impact of the climatic factors that resulted as significant predictors of the farm prevalence.

Spatial analysis showed differences between *T. gondii* and *N. caninum* infections in sheep and goats. Particularly, *T. gondii* infection in goats did not seem to be affected by the geographical location of goat herds. Indeed, no risk clusters were identified for this protozoan. The main reason could lie in the seroprevalences different between sheep and goat. As previously demonstrated by Gazzonis et al. [14], the distribution of infected animals varies among farms according to host species, and at herd level, the seroprevalences were highest in goat than sheep. Goat herds were found to be less different with regard to *T. gondii* herd seroprevalence than sheep herds, and this may have prevented them from highlighting any associations with the study area and analyzed climate variables. Gazzonis et al. [14] suggested that such differences in seroprevalence may be probably explained by a difference in the immune response between goats and sheep, though few studies have beem published on differences in susceptibility to toxoplasmosis of the two species. Also, differences in the biological cycle of *T. gondii* and *N. caninum* may explain the differences found between sheep and goat infection. For example, the predominance of horizontal transmission with respect to vertical transmission, which can be different between *T. gondii* and *N. caninum* in sheep and goats, is still worthy of further investigation. Indeed, while for *T. gondii*, horizontal transmission is suggested as predominant in small ruminants [43], for *N. caninum,* further studies are necessary. Vertical transmission is considered the main mode of *N. caninum* transmission in cattle, but few studies have been conducted on the frequency of this mode of transmission in small ruminants [44,45,46].

Finally, different feeding behavior has been suggested as a variable capable of modulating the risk of acquiring *T. gondii* and *N. caninum* infections [47]. Sheep, which are generally grazers, are more exposed to the risk of getting infected by pathogens found close to the ground compared to goats, which are generally browsers [48]. This is also reflected in higher seroprevalence values in sheep than in goats, even at the individual level [14,15].

The geospatial analysis performed in the present study identified clusters at high or low risk of infection for *T. gondii* and *N. caninum*. In the high-risk areas, preventive measures should be applied to reduce the impact of infections on sheep and goat farms. Particularly, greater attention should be given to the hygienic measures and procedures in farms by, for example, keeping indoor animals by denying access to pasture, denying dogs and cats on the farm and at sites food storage, controlling rodents and other animal pests, and using municipal water instead of surface water [49]. However, the mentioned control measures are not always applicable in the context of sheep and goat breeding, with many family-run farms, or farms with extensive management and access to pasture, or even transhumant flocks.

## 5. Conclusions

In conclusion, the results obtained in the present study revealed a heterogenic distribution of seroprevalence for both *T. gondii* and *N. caninum* in sheep. In this species, indeed, clusters of high and low risk of infection were identified. The considered environmental factors (i.e., annual temperature, rainfall, and their association) were shown to enhance the risk of both infections. On the contrary, no significant cluster resulted for goat farms, and geographical or environmental factors did not seem to be associated with the risk for goats to acquire those infections.

Spatial analysis was confirmed as a useful tool to implement control measures in high-risk areas to prevent the spread of the diseases among small ruminants’ populations. In the spatial analysis, data should always be interpreted considering factors concerning farm management. Indeed, territorial, zootechnical, and economic characteristics may lead to a heterogenous distribution of cases and thus to the calculation of clusters of infections which do not depend on geographical or environmental features, but on the structural characteristics of sampled herds. Moreover, climatic data should be considered in geospatial analyses, since variables such as temperature and rainfall could contribute to maintain and spread the infection in the study areas.

## Figures and Tables

**Table 1:** Possible risk factors associated with higher *Toxoplasma gondii* prevalence in sheep farms using univariate analysis.

Variables	Odds Ratio (OR)	95% Confidence Interval (CI)	*p*-Value
Annual mean temperature (continuous variable)	0.080	0.001–5.959	0.130
Annual rainfall (continuous variable)	0.997	0.992–1.001	0.140

**Table 2:** Risk factors associated with higher *Toxoplasma gondii* prevalence in sheep farms using multivariate multilevel modeling.

Variables	Odds Ratio (OR)	95% Confidence Interval (CI)	*p*-Value
Annual mean temperature (continuous variable)	2.271	1.219–4.232	0.010
Annual rainfall (continuous variable)	1.093	1.022–1.169	0.009
Annual mean temperature/ annual rainfall interaction	0.9991	0.9984–0.9997	0.007

**Table 3:** Possible risk factors associated with higher *Neospora caninum* prevalence in sheep farms using univariate analysis.

Variables	Odds Ratio (OR)	95% Confidence Interval (CI)	*p*-Value
Annual mean temperature (continuous variable)	1.004	0.964–1.046	0.835
Annual rainfall (continuous variable)	1.001	0.997–1.006	0.584

**Table 4:** Risk factors associated with higher *Neospora caninum* prevalence in sheep farms using multivariate multilevel modeling.

Variable	Odds Ratio (OR)	95% Confidence Interval (CI)	*p*-Value
Annual mean temperature (continuous variable)	0.567	0.381–0.844	0.005
Annual rainfall (continuous variable)	0.947	0.908–0.987	0.010
Annual mean temperature/ annual rainfall interaction	1.0006	1.0002–1.001	0.005

**Table 5:** Possible risk factors associated with higher *Neospora caninum* prevalence in goat farms using univariate analysis.

Variables	Odds Ratio (OR)	95% Confidence Interval (CI)	*p*-Value
Annual mean temperature (continuous variable)	0.945	0.880–1.014	0.116
Annual rainfall (continuous variable)	1.105	0.971–1.257	0.130
